# Nonlinear Chemical Process Fault Diagnosis Using Ensemble Deep Support Vector Data Description

**DOI:** 10.3390/s20164599

**Published:** 2020-08-16

**Authors:** Xiaogang Deng, Zheng Zhang

**Affiliations:** College of Control Science and Engineering, China University of Petroleum, Qingdao 266580, China; z18050063@s.upc.edu.cn

**Keywords:** deep learning, ensemble learning, fault detection, support vector data description

## Abstract

As one classical anomaly detection technology, support vector data description (SVDD) has been successfully applied to nonlinear chemical process monitoring. However, the basic SVDD model cannot achieve a satisfactory fault detection performance in the complicated cases because of its intrinsic shallow learning structure. Motivated by the deep learning theory, one improved SVDD method, called ensemble deep SVDD (EDeSVDD), is proposed in order to monitor the process faults more effectively. In the proposed method, a deep support vector data description (DeSVDD) framework is firstly constructed by introducing the deep feature extraction procedure. Different to the traditional SVDD with only one feature extraction layer, DeSVDD is designed with multi-layer feature extraction structure and optimized by minimizing the data-enclosing hypersphere with the regularization of the deep network weights. Further considering the problem that DeSVDD monitoring performance is easily affected by the model structure and the initial weight parameters, an ensemble DeSVDD (EDeSVDD) is presented by applying the ensemble learning strategy based on Bayesian inference. A series of DeSVDD sub-models are generated at the parameter level and the structure level, respectively. These two levels of sub-models are integrated for a holistic monitoring model. To identify the cause variables for the detected faults, a fault isolation scheme is designed by applying the distance correlation coefficients to measure the nonlinear dependency between the original variables and the holistic monitoring index. The applications to the Tennessee Eastman process demonstrate that the proposed EDeSVDD model outperforms the traditional SVDD model and the DeSVDD model in terms of fault detection performance and can identify the fault cause variables effectively.

## 1. Introduction

With the increasing scale and complexity of modern industrial processes, timely fault diagnosis technology is gaining its importance because of the high demands for plant safety and process continuity. Due to the application of the advanced data acquisition and computer control systems, huge volumes of data are collected so that data-driven fault diagnosis methods have been one of the most popular process monitoring technologies in recent years [1,2,3]. Intrinsically, data-driven fault detection can be viewed as one anomaly detection task. As a classic anomaly detection method, support vector data description (SVDD) has received widespread attention in the process monitoring and fault diagnosis field [4,5].

Support vector data description (SVDD) is firstly proposed by Tax and Duin for the one-class classification problem [6,7]. For the nonlinear data, SVDD firstly projects the raw data onto a high-dimensional feature space by the kernel trick and then find a minimal hypersphere to enclose the data samples. Because of its effectiveness in complicated data description, SVDD has obtained extensive applications in the anomaly detection field. For detecting the intrusion behaviors in computer networks, GhasemiGol et al. [8] applied the SVDD to build the ellipsoid boundary of normal behaviors. Sindagi et al. [9] developed the adaptive SVDD for the surface defect detection of organic light emitting diode. Chen et al. [10] proposed the SVDD approach based on spatiotemporal and attribute correlations to detect the anomaly nodes in the wireless sensor networks. In all these anomaly detection tasks, SVDD supposes that most of the training samples are normal and creates a minimized hypersphere to surround these normal samples. The samples apart from the hypersphere are thought to be anomaly data. That means, SVDD belongs to one kind of unsupervised learning algorithm, which only needs the normal samples for model training.

The unsupervised learning characteristic of SVDD is especially suitable to the data-driven fault diagnosis problem. In the industrial processes, most of the collected operating samples are normal while the faulty data are very rare. Even if some fault data exist, they are often unlabeled. Therefore, the statistical process monitoring can be also viewed as the anomaly detection problem. In fact, there have been some scholars applying SVDD algorithm to industrial process monitoring and fault detection. By regarding the process monitoring as one-classification problem, Ge et al. [11] designed a SVDD based batch process monitoring method and indicated that SVDD based fault detection has the advantages of requiring no Gaussian distribution assumption. Jiang et al. [12] combined neighborhood preserving embedding with SVDD to improve chemical process monitoring performance. Huang et al. [13] utilized mutual information technology to distinguish the independent variables and then applied SVDD to build the monitoring model for these independent variables. In order to address the multimodal process monitoring issue, Li et al. [14] designed a weighted SVDD method according to the local density ratio. For monitoring the batch process with time-varying dynamic characteristic, Lv et al. [15] presented one improved SVDD algorithm by integrating the just-in-time learning strategy. Considering the non-Gaussian property of industrial process data, Dong et al. [16] designed the independent component analysis (ICA) based improved SVDD method, which performs SVDD modeling on the non-Gaussian components for sensor fault detection. For dealing with the fault detection of rolling element bearings, Liu et al. [17] proposed a semi-supervised SVDD method to overcome the limitation of labeling samples. Some other related studies can be seen in literature [18,19,20].

Although the present studies demonstrate the practicality of SVDD in some fault diagnosis cases, there are some related problems deserving deep studies. One problem is how to improve the feature extraction capability for the high-dimensional nonlinear chemical process data. The conventional SVDD may not provide accurate data description in the complicated cases. The main reason is its intrinsic shallow learning structure. It is investigated that the SVDD only involves one feature extraction layer based on the kernel function. Therefore, the data description ability of SVDD is limited. In recent years, deep learning theory is thriving in the machine learning and data mining field. Deep neural networks learn the abstract representations of data automatically by multiple feature extraction layers, and have achieved great success in speech recognition, face recognition and drug discovery etc. [21,22,23]. Some initial studies have been performed on the SVDD model [24]. However, how to integrate the deep learning idea with SVDD for fault detection is still unexploited. Another problem is about the fault cause variable location in the SVDD model. This is rarely studied in the SVDD based fault detection model. Once the deep neural network is applied, the improved SVDD model will be more complex and further increase the difficulty of locating the fault cause variables.

Motivated by the above analysis, this paper proposes one ensemble deep SVDD (EDeSVDD) method for nonlinear process fault detection. The contributions of this method are three-fold. (1) we introduce the idea of deep SVDD into the process monitoring field and build one deep SVDD (DeSVDD) based fault detection method for deeper data feature representation. To our best knowledge, there is no studies introducing the fault detection applications of DeSVDD. (2) we present a model ensemble strategy based on Bayesian inference to enhance the monitoring performance of DeSVDD. One problem of DeSVDD based fault detection is that the model structure and the initial weights may affect the model training results. It is difficult to select the optimal model structure and initial parameters in the unsupervised case. So we design the ensemble DeSVDD model by integrating the different models for a holistic monitoring. (3) A novel nonlinear fault isolation scheme based on the distance correlation is designed to identify the possible cause variables. This scheme indicates which original variables are responsible to the occurrence of fault by measuring the nonlinear association. Finally, in order to validate the effectiveness of the EDeSVDD, the applications on the benchmark Tennessee–Eastman (TE) process are illustrated and discussed.

The rest of this paper is organized as follows. Section 2 gives a simple review on the basic SVDD method. Then the proposed EDeSVDD method is clarified in Section 3, which involves the deep model construction, multiple model ensemble, fault variable isolation and the process monitoring procedure. A case study on the TE process is demonstrated in Section 4. Lastly, some conclusions are drawn in Section 5.

## 2. SVDD Method

SVDD is a widely used anomaly detection method. Its main idea is to first map the training data in the original space into a high-dimensional feature space and then seek a minimum-volume hypersphere as a boundary of the training samples [6,7]. The data outside the boundary are detected as the anomaly or faulty samples. The SVDD mathematical description is given as follows.

Given the normal training data set as $X={\left[x_{1}\;x_{2}\;\ldots \;x_{n}\right]}^{T}$ with *n* samples, where $x_{i}\in R^{m}\left(1\leq i\leq n\right)$ is one sample with *m* variables, SVDD projects the original data onto feature space by introducing a nonlinear transformation $\psi (x_{i})$. The training data in the feature space are expressed as:

(1) $$\psi \left(X\right)={\left[\psi \left(x_{1}\right)\;\psi \left(x_{2}\right),\ldots \;\psi \left(x_{n}\right)\right]}^{T}.$$

In the feature space, an optimization objective is designed to find the minimal hypersphere with the center c and the radius *R*, which is written by:

(2) $$\min_{R,\;c,\;\xi_{i}}=R^{2}+\gamma \sum_{i=1}^{n}\xi_{i},$$

(3) $$s.t.\;||\psi \left(x_{i}\right)-c{\left|\right|}^{2}\leq R^{2}+\xi_{i},$$

(4) $$\xi_{i}\geq 0,\;i=1,2,\ldots ,n,$$

where ||·|| represents the Euclidean norm, $\xi_{i}$ is the slack variable allowing a soft boundary, and γ is the trade-off parameter balancing the volume of the hypersphere and the samples out of the boundary.

To solve the above optimization problem, a Lagrange function is constructed as follows:

(5) $$L=R^{2}+\gamma \sum_{i=1}^{n}\xi_{i}-\sum_{i=1}^{n}\alpha_{i}(\xi_{i}+R^{2}-||\psi \left(x_{i}\right)-{c||}^{2})-\sum_{i=1}^{n}\beta_{i}\xi_{i},$$

where $\alpha_{i}>0$ and $\beta_{i}>0$ are the Lagrange multipliers. By the saddle point condition, the SVDD optimization can be reconstructed as the maximization optimization as:

(6) $$\max_{\alpha_{i}}\sum_{i=1}^{n}\alpha_{i}K\left(x_{i},x_{i}\right)-\sum_{i=1}^{n}\sum_{j=1}^{n}\alpha_{i}\alpha_{j}K\left(x_{i},x_{j}\right),$$

(7) $$s.t.\;0\leq \alpha_{i}\leq \gamma ,\;i=1,2,\ldots ,n,$$

(8) $$\sum_{i=1}^{n}\alpha_{i}=1,$$

where $K\left(x_{i},\;x_{j}\right)=\;<\psi \left(x_{i}\right),\;\psi \left(x_{j}\right)>$ is the kernel computation. In this paper, the radial basis kernel function is applied as [4]:

(9) $$K\left(x_{i},\;x_{j}\right)=exp\left(-\frac{\left|\right|x_{i}-x_{j}{\left|\right|}^{2}}{\sigma }\right),$$

where σ is the width of Gaussian kernel.

Equations (6)–(8) describe a standard quadratic optimization problem, which can be solved by many methods such as the sequential minimal optimization. The solution leads to a series of $\alpha_{i}$. Usually, $\alpha_{i}=0$ means the corresponding sample is inside of the hypersphere, while the sample with $0<\alpha_{i}<\gamma$ is on the hypersphere boundary, which is called support vector (SV). When $\alpha_{i}=\gamma$, the corresponding samples fall outside of hypersphere, which are called unbounded support vectors (USVs) [5]. The hypersphere center c can be computed by:

(10) $$c=\sum_{i=1}^{n}\psi \left(x_{i}\right)\alpha_{i},$$

while the radius *R* can be obtained by computing the distance between the support vectors and the center, shown as:

(11) $$R=||\psi \left(x_{i}\right)-c\left|\right|,\;x_{i}\in SV.$$

For any test vector $x_{t}$, the squared distance to the center c, denoted as $D_{t}$, can be used as a monitoring index for judging if the test vector belongs to an anomaly point. Its expression is given by [15]:

(12) $$D_{t}=||\psi \left(x_{t}\right)-c{\left|\right|}^{2}.$$

By combining Equation (10), the SVDD monitoring index is constructed for fault detection, which is rewritten as:

(13) $$D_{t}=K\left(x_{t},x_{t}\right)-2\sum_{i=1}^{n}\alpha_{i}K\left(x_{i},x_{t}\right)+\sum_{i,j=1}^{n}K\left(x_{i},x_{j}\right).$$

If $D_{t}\leq R$, the corresponding vector $x_{t}$ is classified as the normal sample. Otherwise, it is regarded as the abnormal sample. By comparing the $D_{t}$ with *R*, we can judge the condition of process data.

**Figure 1:**
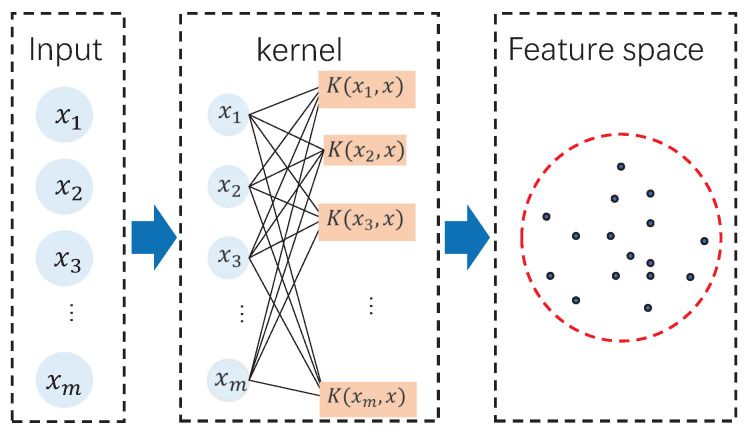
Support vector data description (SVDD) schematic.

In the SVDD algorithm, the feature extraction and data description procedure can be depicted as shown in Figure 1. By investigating the SVDD procedure, there is only one feature extraction layer based on the kernel function. The kernel function maps the original data into the high-dimensional feature space and then in the feature space, the one-class data description is performed by the optimization in Equations (2)–(4). Therefore, it is clear that the feature extraction is shallow since only one feature layer is involved. Although the kernel function is strong, it is still difficult to describe the complicated nonlinear data relationship. To mine the data feature sufficiently, it is necessary to perform deep learning. This motivates the presentation of deep SVDD.

## 3. Fault Diagnosis Method Based on Ensemble Deep SVDD

### 3.1. Deep SVDD Model Construction

**Figure 2:**
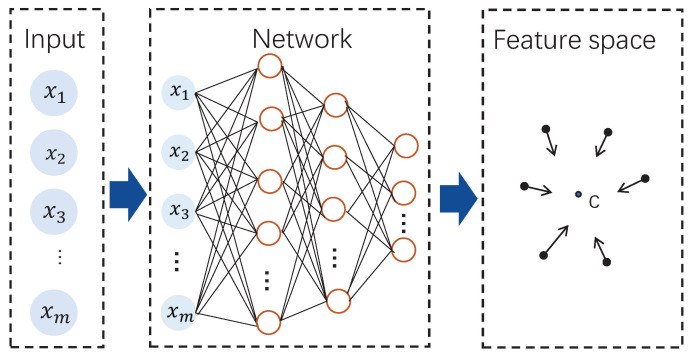
Deep SVDD schematic.

The deep learning based SVDD model was firstly discussed in Ruff et al.’s work [24,25], which performs deep network training with optimizing a data-enclosing hypersphere and achieves the successful applications the image processing field. In this paper, we design the deep SVDD (DeSVDD) based fault detection method. A DeSVDD framework is shown as Figure 2. Similar to kernel-based SVDD, DeSVDD aims to find a hypersphere in the feature space so that the hypersphere with the minimal radius can surround all normal samples. The biggest difference is that DeSVDD automatically learns useful data feature representations by multiple feature layers not by only one feature layer.

For the training data X with *n* samples $\left\{x_{i}\in R^{m},i=1,2,\ldots ,n\right\}$, the multi-layer feature extraction procedure is denoted as $\Phi (x_{i};W)$, where Φ(.) represents the nonlinear mapping procedure in deep network, and W is the network weight set. By the deep data mining, the final feature can be written as:

(14) $$\Phi \left(X\right)={\left[\Phi \left(x_{1},W\right)\;\Phi \left(x_{2},W\right)\;\cdots \;\Phi \left(x_{n},W\right)\right]}^{T}.$$

The goal of deep SVDD is designed as Equation (15), which is to minimize the volume of the hypersphere surrounding the data Φ(X) by learning the network parameter W. At the same time, the weight matrix is used as the regularization item [24].

(15) $$\min_{W}\frac{1}{n}\sum_{i=1}^{n}∥\Phi \left(x_{i};W\right){-c∥}^{2}+\frac{\lambda }{2}\sum {∥W∥}_{F}^{2},$$

where λ is the trade-off parameter.

In this optimization objective, the training samples are mapped to the features close to center c as possible. Considering that all given samples belongs to the normal class, the average distance from the hypersphere center is penalized instead of allowing some points to fall outside the hypersphere.

Usually, we can learn the network parameter set W by the stochastic gradient descent (SGD) algorithm, which can be performed in some mature software tools such as Tensorflow. The SGD has the advantages of high training efficiency with large data sets. However, in the deep SVDD training procedure, some remarks should be noted [24].

**Remark** **1.** *The determination of hypershphere c should avoid the trivial solutions. From Equation (15), it is seen that if c is set to zero, the solution of W may be solved as the all-zero weight set so that $∥\Phi (x_{i};W)-c∥=0$. That means we can not obtain the valuable feature information. To avoid this, the c can be empirically fixed to the mean of the network outputs, obtained by the pre-training based on the autoencoder network.*

**Remark** **2.** *The hidden layer activation functions should have no bias item b to ensure the non-trivial solutions. If the bias items are applied in the activation functions, the optimization may lead to the all-zero W. This would not reflect the features of the input data, but only trains the bias items so that $∥\Phi (x_{i};W)-c∥=0$ with W=0.*

**Remark** **3.** *The unbounded activation function such as ReLU should be applied to avoid the hypersphere collapse. If the deep SVDD network is equipped with the bounded activation function, it may be saturated for all inputs.*

For the testing sample $x_{t}$, its distance to the center c is used to evaluate the anomaly degree of the sample. For convenience, a monitoring index is defined the squared distance as:

(16) $$D_{t}=||\Phi \left(x_{t};W^{*}\right)-c{\left|\right|}^{2},$$

where $W^{*}$ is the weights of the trained network. Since there is no defined radius in this method, we have to find another way to set the detection threshold. Here we use the kernel density estimation (KDE) to obtain the 99% confidence limit of the monitoring statistic $D_{t}$, which is applied as the fault detection threshold. For given testing sample, it will be considered to be fault when the corresponding index $D_{t}$ exceeds its 99% confidence limit.

KDE is an effective non-parametric tool for estimating the probability density function (PDF) of a random variable [26]. For the normal samples $x_{1}\;x_{2}\;\cdots \;x_{n}$, we can compute its monitoring indices $D_{1},\;D_{2},\;\cdots ,\;D_{n}$, which can be viewed as the random sampling points of monitoring index $D_{t}$. The PDF of $D_{t}$ can be estimated as [26,27]:

(17) $$f\left(D_{t}\right)=\frac{1}{nw}\sum_{i=1}^{n}g\left(\frac{D_{t}-D_{i}}{w}\right),$$

where *w* is the smoothing parameter, g(.) is the kernel function for density estimation. With the estimated PDF and the given confidence level α, we can determine the confidence limit $D_{lim}$ for the index $D_{t}$ by solving the following expressions [27]:

(18) $$\int_{-\infty }^{D_{lim}}f\left(D_{t}\right)d\left(D_{t}\right)=\alpha .$$

By the KDE technique, we get a reasonable threshold for the monitoring index $D_{t}$. If $D_{t}>D_{lim}$, that means a fault is detected with the confidence level of α. In this paper, α is set to 99%.

In the SVDD model, the anomaly detection based on *R* is dependent on the pre-determined parameter γ. With different γ, the radius *R* has different values. This way it is difficult to explain the probabilistic meaning of the data description. Therefore, even for the SVDD model, KDE is also very suitable and adopted in this paper.

### 3.2. Multiple Deep Models Ensemble with Bayesian Inference Strategy

Although the DeSVDD provides the potential to extract the more meaningful features for fault detection, its shortcomings should also be noted. One is about the model structure determination. That is, how many layers should be used for the given data set and how many nodes should be used in each layer? In fact, this is an open problem in the deep learning category. Up to now, the present deep learning studies do not give a conclusion about this issue. In many supervised learning cases, researchers can adopt the trial-and-error strategy to choose a reasonable but not optimal network structure. Another problem involves the weight initialization. As the deep SVDD network adopt the stochastic training algorithm, the trained models may be different for each running. This is not a serious problem in the supervised case as the users can adopt the best one according to the classification performance. However, the DeSVDD is intrinsically dealing with one unsupervised problem and it is difficult to determine the optimal initial parameters based on the training data.

**Figure 3:**
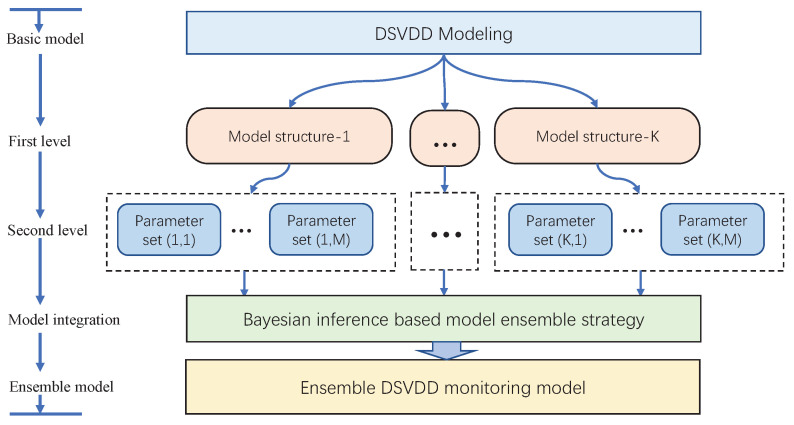
Ensemble Deep SVDD schematic.

Considering the unsupervised property of the DeSVDD, the ensemble learning strategy is applied to build the improved DeSVDD method for dealing with these two above issues. In this method, a two-level ensemble framework is designed, which is shown in Figure 3. At first level, some different typical network structures are applied, while different initial parameters are adopted for each structure at the second layer. By using the multiple DeSVDD models, the uncertainty of process monitoring is eliminated and a comprehensive monitoring model is constructed.

In the Figure 3, there are a series of DeSVDD models available. If the first level designs *K* groups of the model structures and the second level provides *M* groups of the initial parameter sets for each structure, the total number of DeSVDD models *B* is computed as B=KM. In order to merge all the DeSVDD models for a holistic monitoring, Bayesian inference theory is applied to design an ensemble strategy [28,29].

For the testing vector $x_{t}$, the *b*-th (1≤b≤B) DeSVDD model has the monitoring statistic $D_{t}^{b}$ with the corresponding detection threshold as $D_{lim}^{b}$. By the Bayesian inference theory, the fault probability $P^{b}\left(F|x_{t}\right)$ induced by the monitoring index $D_{t}^{b}$ when the vector $x_{t}$ occurs is developed as:

(19) $$P^{b}\left(F|x_{t}\right)=\frac{P^{b}\left(x_{t}|F\right)P^{b}\left(F\right)}{P^{b}\left(x_{t}|N\right)P^{b}\left(N\right)+P^{b}\left(x_{t}|F\right)P^{b}\left(F\right)},$$

where $P^{b}\left(F\right)$ and $P^{b}\left(N\right)$ represent the prior fault probability and the prior normal probability, respectively. If the confidence level of the normal training data is set as α, there are the results of $P^{b}\left(F\right)=1-\alpha$ and $P^{b}\left(N\right)=\alpha$. $P^{b}\left(x_{t}|F\right)$ represents the prior fault probability of the sample $x_{t}$, while $P^{b}\left(x_{t}|F\right)$ denotes the prior normal probability. They are computed by [28]:

(20) $$P^{b}\left(x_{t}|F\right)=exp\left(-\frac{D_{lim}^{b}}{D_{t}^{b}}\right),$$

(21) $$P^{b}\left(x_{t}|N\right)=exp\left(-\frac{D_{t}^{b}}{D_{lim}^{b}}\right).$$

By Equation (19), the fault probabilities for the DeSVDD models are obtained. The next is to combine them for a holistic index. Here we apply the conditional fault probability to weight the Equation (19) so that the holistic monitoring index $BD_{t}$ is expressed by:

(22) $$BD_{t}=\sum_{b=1}^{B}P^{b}\left(F|x_{t}\right)w^{b}\left(x_{t}\right),$$

where $w^{b}\left(x_{t}\right)$ is the fusion weight, which can be computed by the prior fault probability as:

(23) $$w^{b}\left(x_{t}\right)=\frac{exp(P^{b}\left(x_{t}|F\right)/2)}{\sum_{l=1}^{B}exp\left(P^{l}\left(x_{t}|F\right)/2\right)},$$

where $BD_{t}$ should be under the confidence level α under the normal condition. Therefore, its detection threshold $BD_{lim}$ can be set to be α. However, the detection threshold can also be estimated by KDE method. In this paper, we adopt the latter.

So far, we complete the modeling process of the EDeSVDD algorithm. By applying ensemble learning theory, EDeSVDD can provide a more reasonable monitoring on the complicated industrial processes.

### 3.3. Fault Variable Isolation Using Distance Correlation

The monitoring index in Equation (22) can indicate if some fault occurs but cannot determine which variable causes the fault. In order to provide the useful fault repair information to engineers, it is necessary to isolate the cause variables. In the traditional data-driven fault diagnosis field, contribution plot is one common way, which determines the faulty variables by the linear correlation analysis [30]. However, fault isolation in the EDeSVDD model is a challenging task since the complicated nonlinear transformations are involved in the deep learning neural network. To handle this problem, we propose a novel faulty variable isolation method based on the distance correlation, which is capable of measuring the nonlinear dependency between the original monitored variables and the holistic monitoring index.

The traditional linear correlation relationship is measured by the Pearson correlation coefficient. For two given random variables *y* and *z*, their Pearson correlation coefficient is defined by:

(24) $$\rho_{p}\left(y,z\right)=\frac{\sum_{i=1}^{n}\left(y_{i}-\bar{y}\right)\left(z_{i}-\bar{z}\right)}{\sqrt{\sum_{i=1}^{n}{\left(y_{i}-\bar{y}\right)}^{2}\sum_{i=1}^{n}{\left(z_{i}-\bar{z}\right)}^{2}}},$$

where $y_{i}$ and $z_{i}$ are the *i*-th sample points of the variable *y* and *z*, respectively, $\bar{y}$ and $\bar{z}$ are the corresponding mean values.

Pearson coefficient is based on the process linear assumption and may not perform well in the nonlinear chemical processes. Therefore, this section introduces the distance correlation for fault isolation. Distance correlation, firstly proposed by Szekely et al. [31], is more suitable way to investigate the nonlinear variable relationships. It is designed based on the characteristic function of sets of random variables and can give the true relationship measure. The distance correlation coefficient of two random variables *y* and *z* can be expressed by [32,33]:

(25) $$\rho_{d}\left(y,z\right)=\sqrt{\frac{\sum_{i,j=1}^{n}{\bar{U}}_{i,j}{\bar{V}}_{i,j}}{\sqrt{\sum_{i,j=1}^{n}{{\bar{U}}_{i,j}}^{2}\sum_{i,j=1}^{n}{{\bar{V}}_{i,j}}^{2}}}},$$

where $\bar{U}$ and $\bar{V}$ are the centered distance matrices, which are defined by:

(26) $$\bar{U}=U-I_{n\times 1}\times u^{c}-u^{r}\times I_{1\times n}-u^{rc},$$

(27) $$\bar{V}=V-I_{n\times 1}\times v^{c}-v^{r}\times I_{1\times n}-v^{rc},$$

where U and V have their (i,j)-th elements as $U_{i,j}=\left|\right|y_{i}-y_{j}\left|\right|$, and $V_{i,j}=\left|\right|z_{i}-z_{j}\left|\right|$, respectively. $u^{c}$ and $v^{c}$ represent the column mean vector of U and V, respectively. $u^{r}$ and $v^{r}$ represent the row mean vector of U and V, respectively. $u^{rc}$ and $v^{rc}$ are the grand means.

Based on the distance correlation coefficient, we can obtain the associations between the monitoring index and the original variables. For the variable $x_{i}$, its fault association degree is defined as the normalized distance correlation, expressed by:

(28) $$FAD_{i}=\frac{\rho_{d}\left(BD_{t},x_{i}\right)}{max\{\rho_{d}\left(BD_{t},x_{i}\right),1\leq i\leq m\}}.$$

The value of $FAD_{i}$ ranges from 0 to 1. The variable corresponding to $FAD_{i}=1$ indicates the largest fault association degree, and the other variables with high $FAD_{i}$ values are also related to the fault. On the contrary, if $FAD_{i}$ is close to 0, that means the variable $x_{i}$ is under the normal condition.

### 3.4. Process Monitoring Procedure

Process monitoring based on the EDeSVDD mehod can be divided into two stages: offline modeling and online monitoring. In the offline modeling stage, the normal data are collected and trained for the EDeSVDD modeling, while in the online monitoring stage, the new data are collected to project onto the built model and the monitoring index is computed for process condition judgement. The specific steps are as follows:

*Offline modeling stage:*
  1. Collect the historical normal data and divide them into the training data set and validating data set;
  2. Normalize all the data sets by the mean and variance of the training data set;
  3. Apply the normal data set to pretrain the KM deep SVDD networks with autoencoder to determine the center vector;
  4. Train the multiple deep SVDD networks as the basic monitoring sub-models.
  5. Compute the monitoring indices $D_{t}^{b}$ of the validation data and determine the confidence limits $D_{lim}^{b}\left(1\leq b\leq KM\right)$ using KDE method;
  6. Construct the ensemble statistic $BD_{t}$ by Bayesian fusion strategy, and calculate its detection threshold $BD_{lim}$.
*Online monitoring stage:*
  1. Collect testing sample and normalize it with the mean and variance of the training data set;
  2. Project the normalized data onto each deep SVDD model and obtain its monitoring indices $D_{t}^{b}$;
  3. Compute the ensemble index $BD_{t}$ and compare it with the threshold $BD_{lim}$. If $BD_{t}>BD_{lim}$, that means one fault occurs. Otherwise, the process is in the normal status.
  4. When a fault is detected, the fault isolation map is built to identify the fault cause variables.

## 4. Case Study

### 4.1. Process Description

**Figure 4:**
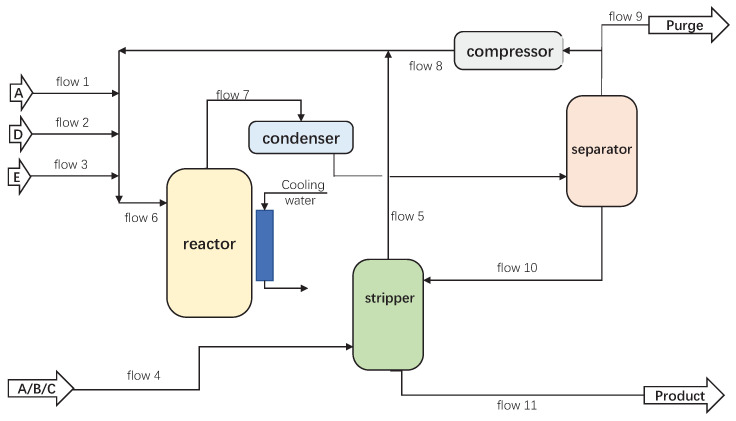
Flowchart of the Tennessee–Eastman (TE) process.

The Tennessee–Eastman (TE) process was applied to evaluate the proposed method. This process is from a real chemical process and has been one benchmark system used to test the different control and diagnosis methods [28,29,34,35,36,37]. This process simulator was firstly created by Downs and Vogel [38] and the corresponding data can be downloaded from the website. The process flowchart is shown in Figure 4, which included five major operation units: a reactor, a condenser, a recycle compressor, a separator, and a stripper [39]. The reactants A, C, D and E and the inert B were fed for the irreversible and exothermic first-order reactions. The output flow of reactor was cooled by the condenser and then goes into the separator. The vapor from the separator returned to the reactor with the use of the compressor, while the liquid from the separator flowed into the stripper, which brought the products G and H, and the byproduct F.

This process had 41 measurement variables and 12 operational variables. A total of 52 variables were involved in fault diagnosis and 21 fault patterns are designed for fault diagnosis method testing. The detailed fault descriptions are listed in Table 1. For the normal operational condition, two data sets including one 960-sample set and one 500-sample set were generated. For each fault pattern, a total of 960 samples were simulated where the fault was introduced after the 160-th sample.

### 4.2. Results and Discussions

#### 4.2.1. Fault Detection

Three methods of SVDD, DeSVDD, and EDeSVDD were applied to detect the faults. The SVDD parameters were set as σ=50, γ=1. The trade-off parameter of DeSVDD was set as $\lambda =10^{-6}$. In the deep network, an improved ReLU activation function called ELU was applied. ReLU is a piecewise linear function which transforms all negative values into 0 while the positive values remain unchanged. The ReLU operation is also called unilateral suppression, which makes the network sparse to alleviate the possible overfitting problem. More details about ReLU activation function can be seen in the literature [40]. ELU function further enhanced the ReLU function by defining the non-zero output for the negative input. For the DeSVDD, two feature extraction layers were used and the corresponding nodes were set to the 70% of the previous layer orderly. That means, the DeSVDD had the layer structure of 52-36-26. In the EDeSVDD, two model structures were applied, which are both the two-hidden layer network. The first model structure was the layer node numbers of 52-36-26, while the second had the nodes of 52-47-42, which means a 90% node setting rule. For each model structure, it was run 10 times with different initial parameters. Thus, the EDeSVDD involved 20 sub-models. For all these methods, two indices including fault detection rate (FDR) and false alarming rate (FAR) were applied to evaluate the monitoring performance. The former is the ratio of the number of fault samples over the threshold to the total fault sample number, while the latter is the percentage of the normal sampler exceeding the threshold over the total normal samples.

**Figure 5:**
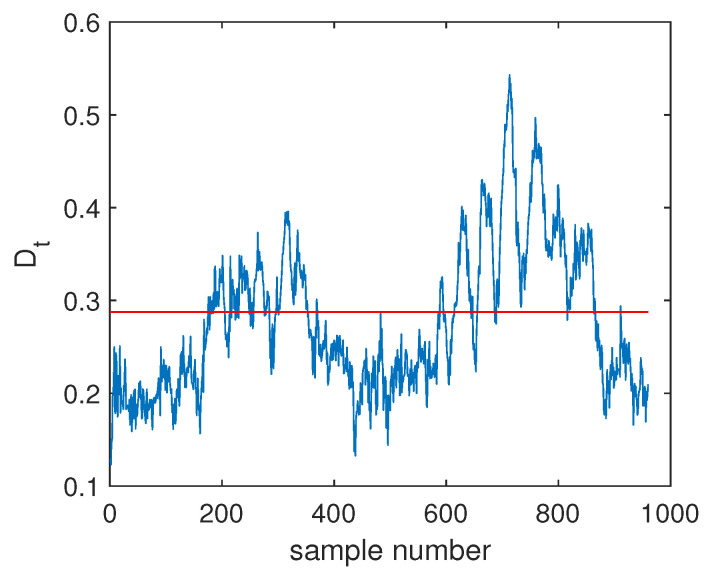
Fault detection chart of fault F10 based on SVDD.

**Figure 6:**
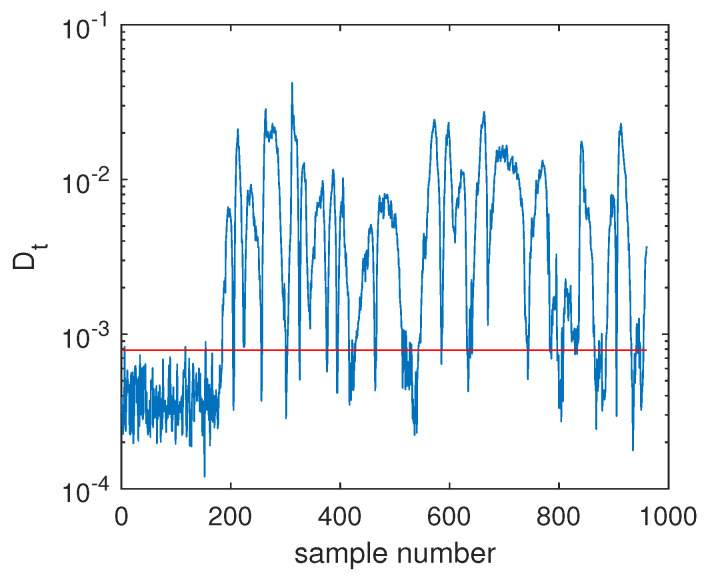
Fault detection chart of fault F10 based on deep SVDD (DeSVDD).

**Figure 7:**
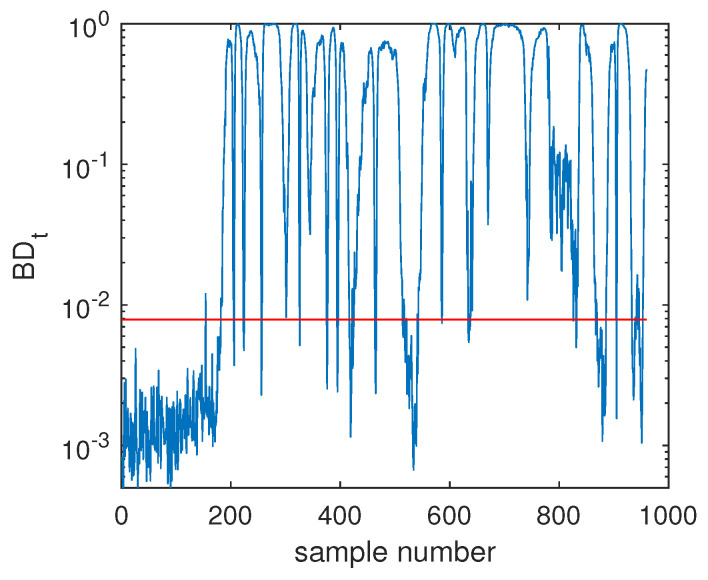
Fault detection chart of fault F10 based on one ensemble deep SVDD (EDeSVDD).

The fault F10 was taken as the first example, and its monitoring charts of three methods are listed in Figure 5, Figure 6 and Figure 7. When the SVDD method was applied, this fault could not be detected effectively. By Figure 5, it is seen that many fault samples were mis-detected by the $D_{t}$ index of SVDD. The FDR of SVDD was a poor 48%. With the use of DeSVDD, the fault samples were detected with a higher FDR of 79.63%. From the DeSVDD monitoring chart in Figure 6, it is seen that more faulty samples were detected. It should be noted that this result was not stable. If the initial parameter set was changed, the monitoring performance may also have been changed. To overcome this shortage, the EDeSVDD model was introduced which integrated a lot of sub-models. Its monitoring chart is shown in Figure 7, where more fault samples exceeded the detection threshold. The FDR of EDeSVDD was 87%, which was higher than the results of SVDD and DeSVDD. Therefore, the EDeSVDD method had a better fault detection performance when the fault F10 occurred.

**Figure 8:**
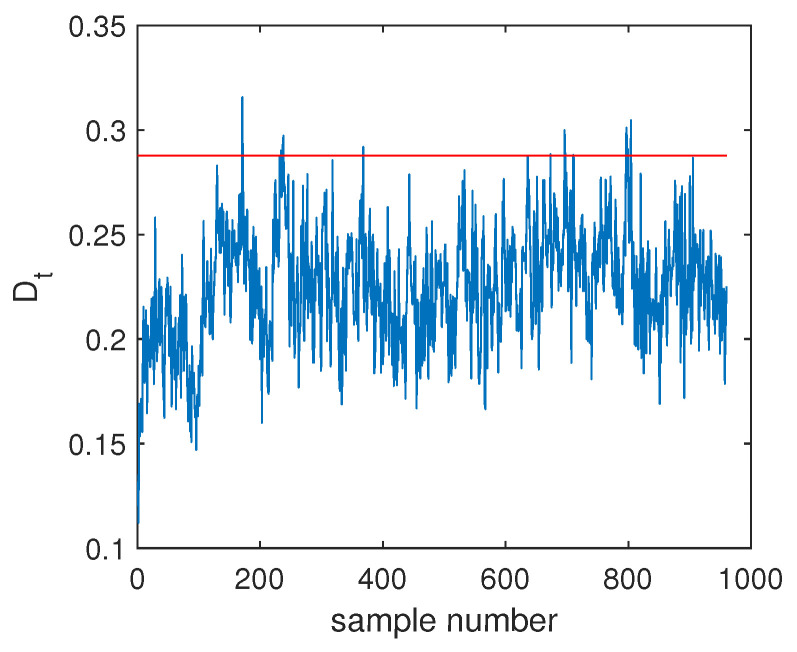
Fault detection chart of fault F19 based on SVDD.

**Figure 9:**
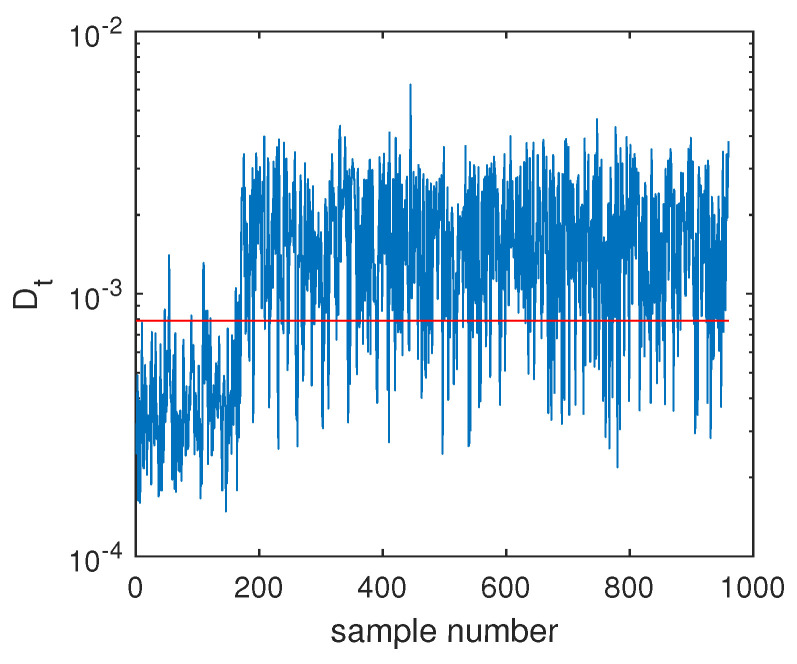
Fault detection chart of fault F19 based on DeSVDD.

**Figure 10:**
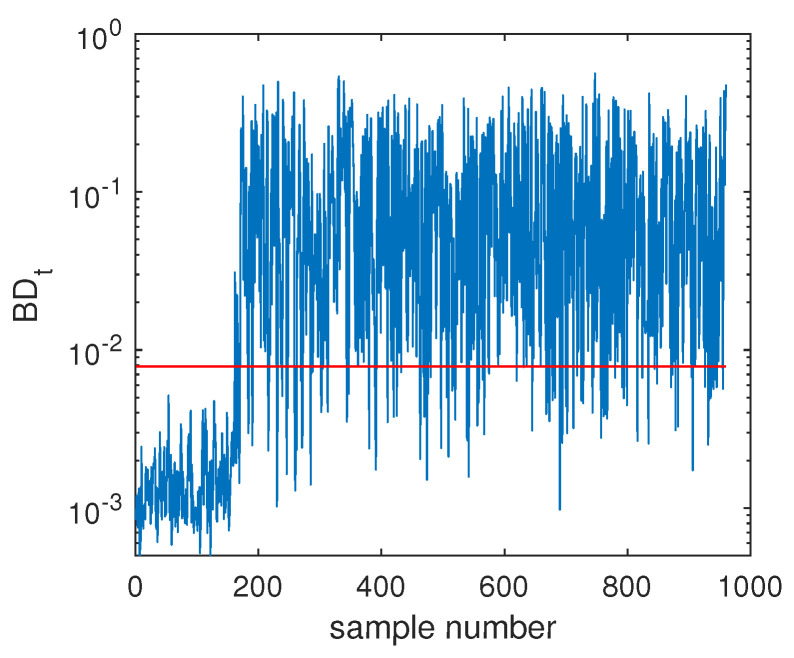
Fault detection chart of fault F19 based on EDeSVDD.

Another illustrated example is Fault F19, which was one unknown small-amplitude fault. Regarding this fault, the SVDD monitoring chart is shown in Figure 8, which indicates that SVDD model could not detect this fault totally. There were no any clear signs for this fault and the fault detection rate was only 1.75%. With the deep feature extraction, the DeSVDD model gave an obviously better result, which is plotted in Figure 9. Almost half of the fault samples went beyond the confidence limit and the FDR was 74.5%. With a comprehensive modeling procedure, EDeSVDD provided a stronger monitoring performance as shown in Figure 10. When the EDeSVDD model was used to monitor this fault, it led to the FDR of 85.88%. The monitoring results on the fault 19 further verified the effectiveness of the proposed method.

**Figure 11:**
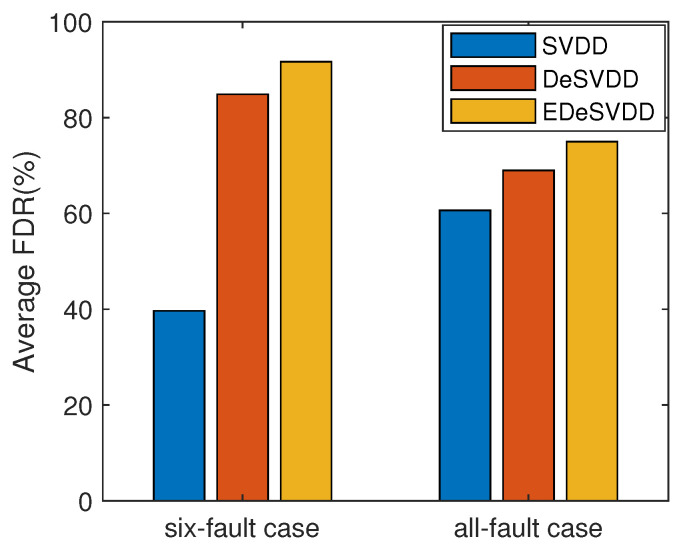
Comparison of average FDR.

A complete FDR comparison of three methods is listed in Table 2. In this table, SVDD is the basic state-of-art model. The monitoring results of DeSVDD method are the statistical indices (i.e., mean and standard deviation) of multiple runnings. Here two model structures including 52-36-26 and 52-47-42 were considered and each model was run 10 times. The average FDR was given with the corresponding standard variation in the Table 2. EDeSVDD was the ensemble model of two model structures with the respective 10 training cycles. To evaluate the stability of results, the EDeSVDD model was also run 10 times. By this table, it is seen that the faults F1, F2, F6, F8, F12, F13, F14, and F18 had similar high FDR values, while the faults F3, F9 and F15 had similar low FDR values for all these methods. These three faults only involved the weak process changes and were difficult to detect in many algorithms. Some specific methods have been studied to deal with these three faults [37] and this paper does focus on them. In regard to the faults F5, F10, F16, F17, F19, and F20, the DeSVDD method was superior to the basic SVDD model, while the EDeSVDD further outperformed the DeSVDD model clearly. There was a special phenomenon for the faults F4, F7, F11 and F21. For these four faults, DeSVDD did worse than the SVDD model. These results demonstrate that the deep model is not almighty. As the deep learning theory is still in research, we have no conclusion about how to select an optimal deep model for the given data learning task. It is also seen that when the ensemble learning strategy is applied, the EDeSVDD model can achieve very clear improvements and may further outperform the basic SVDD model. For example, in the case of fault F21, the SVDD FDR was 37.38%, while the average FDR of DeSVDD was 36.70%. By applying the ensemble model, EDeSVDD enhanced the FDR to 45.08%. On the whole, the mean FDRs of these three methods were 60.65%, 68.99%, and 74.97%, respectively. Compared to the SVDD model, the DeSVDD model prompted the FDR with about 8%. Furthermore, EDeSVDD model had a 8.38% higher FDR than DeSVDD. Along with the improvement of the mean FDR, the standard deviation of EDeSVDD FDR decreased to 1.08% compared to the 3.99% of DeSVDD. That shows EDeSVDD has a higher detection rate with lower performance fluctuations. A comparison of average FDR is shown in Figure 11. For the six fault cases, that means the faults F5, F10, F16, F17, F19, and F20, the DeSVDD and EDeSVDD methods outperformed the SVDD significantly. Even considering all these faults, the proposed methods still had apparent advantages in terms of the fault detection performance.

Besides the fault detection performance, the false alarming performance is also one important evaluation aspect. In all the tested fault data sets, the first 160 samples belonged to the normal condition. We computed the false alarming rate (FAR) on these samples and tabulated in the Table 3. By this table, the SVDD had the highest average FAR as 1.88%. The DeSVDD average FAR was lower, which is 1.08%, but its standard deviation was 0.94%. That means some DeSVDD models may have had a higher or lower FAR. The EDeSVDD model had the lowest average FAR as 0.87% with the smaller standard deviation 0.64%. In view of the FAR index, the proposed EDeSVDD method did best.

In this section, three methods of SVDD, DeSVDD, EDeSVDD were applied for fault detection and their characteristics are summed up as follows. For the traditional SVDD method, it used a shallow learning model with only one kernel mapping layer. This method had no random weights, so ensemble learning was not involved. As to the DeSVDD method, it applied the deep learning model with multiple feature mapping layers. However, DeSVDD may lead to unstable monitoring results due to different random weight settings. For the EDeSVDD, it combined the deep learning and ensemble learning to overcome the disadvantages of these two mentioned methods. It should be noted that the EDeSVDD method had the highest model complexity. This is understandable in view of the no free lunch theorem. For achieving better monitoring performance, a more complicated model is needed.

#### 4.2.2. Fault Isolation

**Figure 12:**
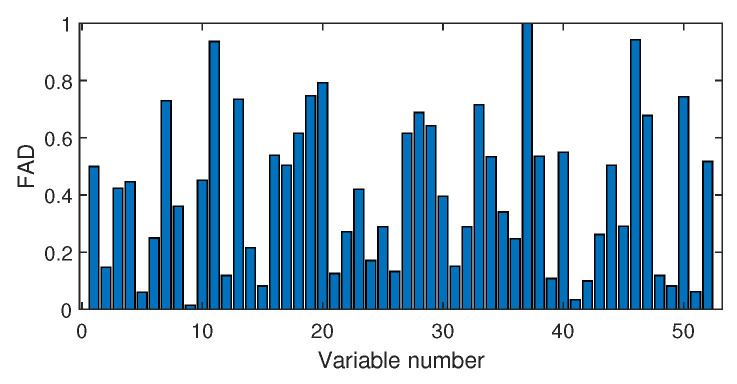
Fault isolation results for fault F10 based on Pearson correlation.

**Figure 13:**
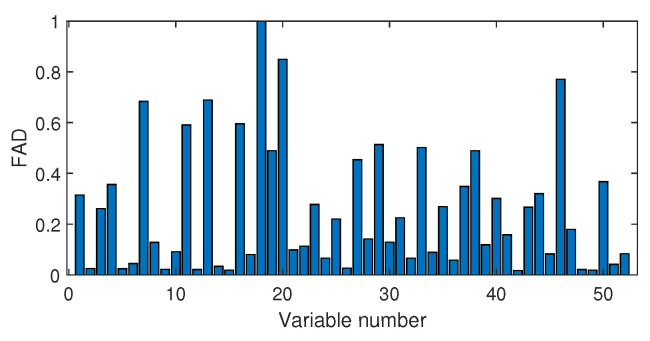
Fault isolation results for fault F10 based on distance correlation.

**Figure 14:**
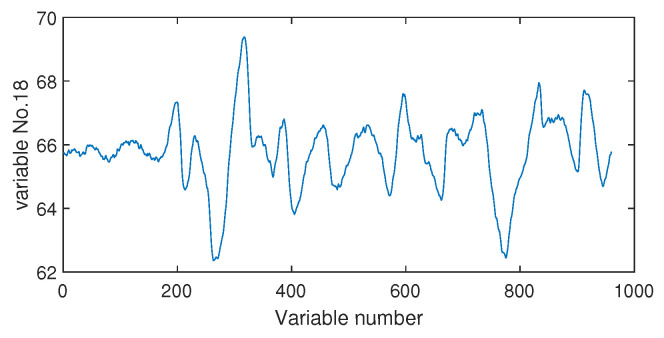
The variable trend (No. 18) when fault F10 occurs.

**Figure 15:**
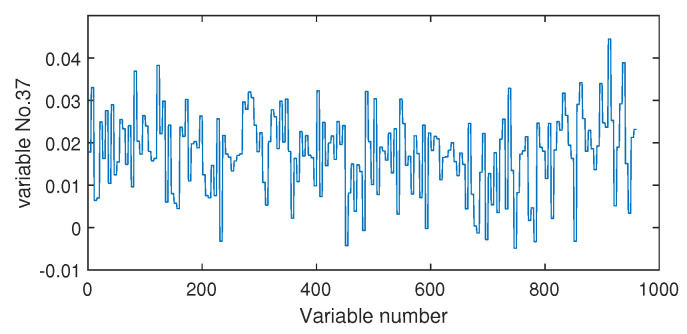
The variable trend (No. 37) when fault F10 occurs.

Next, we discuss the results of fault isolation. Fault F10 is taken as one example. When this fault occurred, we collected the corresponding fault data and computed the fault association degree with the monitoring index by the Pearson correlation $\rho_{p}$ and the distance correlation $\rho_{d}$, respectively. The fault isolation map using the Pearson correlation is given in Figure 12. By this figure, many variables such as variable Nos. 22, 37, 46, and 50 were all with high FAD values. In fact, a total of 14 variables had a fault association degree larger than 0.6. It was difficult for engineers to locate the real cause variables. By contrast, the distance correlation gave a better result in Figure 13. Only six variables had the large FAD values beyond 0.6. Among these six variables, the 18th variable, that corresponded to the stripper temperature, was the most significant fault cause variable. By mechanical analysis, fault F10 was triggered by the change of the stripper feed temperature, which had the consequent influence on the stripper temperature. This can be verified by the variable trend plotted in Figure 14. Therefore, once the stripper temperature was located, the real fault cause could be easily found. The fault isolation map in Figure 12 indicates the 37th variable as the most possible cause, which corresponded to the component D percentage in the product flow. In fact, this variable was not influenced seriously by Figure 15. So, the mechanical analysis of this fault demonstrated that the fault isolation map based on the distance correlation did better.

**Figure 16:**
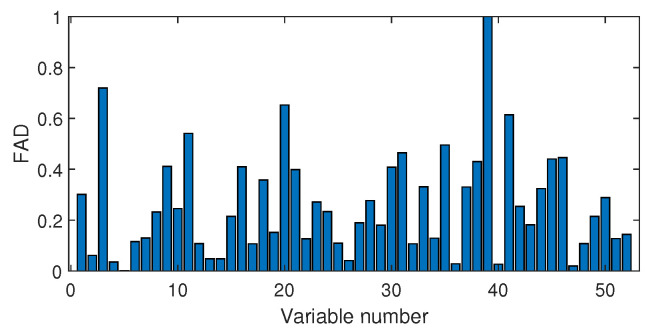
Fault isolation results for fault F19 based on Pearson correlation.

**Figure 17:**
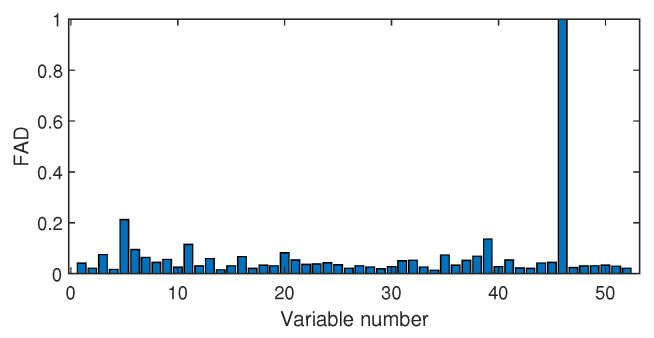
Fault isolation results for fault F19 based on distance correlation.

**Figure 18:**
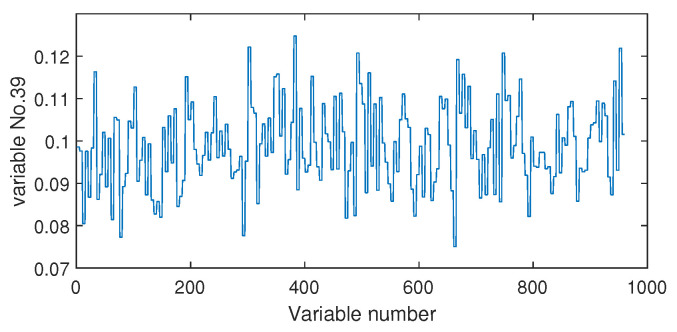
The variable trend (No. 39) when fault F10 occurs.

**Figure 19:**
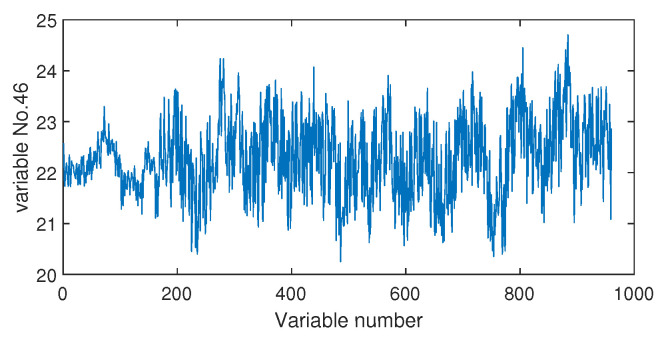
The variable trend (No. 46) when fault F10 occurs.

The fault isolation results of fault F19 are shown in Figure 16 and Figure 17. By Figure 16 obtained by the Pearson correlation analysis, the No. 39 variable was located as the fault cause variable, which had the largest fault association degree. This variable was the component F percentage in the product flow. As one quality variable, it may have been the fault result but could not be the fault source. Its trend is plotted in Figure 18, which shows no significant change occurred. When the distance correlation analysis was applied, the fault isolation map, given in Figure 17, brought a very clear scene, which obviously indicated the No. 46 variable as the fault cause. We plotted the variable trend in Figure 19 and observed that this variable had the larger fluctuations after the 160-th sample. As the fault F19 was an unknown fault, it was impossible to verify our analysis by process mechanism. However, the variable changing trend plots demonstrated that the distance correlation analysis could provide more effective fault isolation compared with the Pearson correlation analysis.

## 5. Conclusions

In order to provide effective monitoring on the chemical processes, this paper designs an improved SVDD based fault detection method by combining the deep learning and ensemble learning. Deep learning is applied to present a deep feature description for the SVDD modeling, while ensemble learning is utilized to overcome some essential disadvantages of deep neural networks. At the same time, a fault isolation map based on the distance correlation is applied to locate the fault cause variables. We test the proposed method on the benchmark TE process. The application results show that the deep learning is effective and the DeSVDD model can improve the FDR of the basic SVDD model for most of the fault cases, and the EDeSVDD model can provide a better monitoring performance with the higher FDR and the lower FAR. However, some problems deserve the further studies. One is about the declining performance in some fault cases. Although DeSVDD outperforms the SVDD in general, it does worse in some cases. A possible reason involves the optimal deep model. To get a more rational reason and further give some viable solution is one valuable topic.

## Figures and Tables

**Table 1:** Process faults of the TE process.

No.	Description
F1	Step change of A/C feed ratio at flow 4
F2	Step change of B composition at flow 4
F3	Step change of D feed temperature at flow 2
F4	Step change of reactor cooling water inlet temperature
F5	Step change of condenser cooling water inlet temperature
F6	Step change of A feed loss at flow 1
F7	Step change of C header pressure loss-reduced availability at flow 4
F8	Random variation of A, B, C feed composition at flow 4
F9	Random variation of D feed temperature at flow 2
F10	Random variation of C feed temperature at flow 4
F11	Random variation of reactor coolant inlet temperature
F12	Random variation of condenser coolant inlet temperature
F13	Slow drift of reaction kinetics
F14	Reactor cooling water valve sticking
F15	Condenser cooling water valve sticking
F16–F20	Unknown faults
F21	The valve for flow 4 sticking

**Table 2:** Fault detection rates (FDRs)(%) of the TE process faults by the SVDD, DeSVDD and EDeSVDD methods.

No.	SVDD	DeSVDD	EDeSVDD
F1	99.38	99.49 ± 0.11	99.59 ± 0.06
F2	98.5	97.89 ± 0.44	98.29 ± 0.08
F3	4.13	2.23 ± 0.83	2.40 ± 0.59
F4	53.5	22.71 ± 15.62	43.26 ± 10.51
F5	27.75	99.94 ± 0.08	100.00 ± 0.00
F6	100	100.00 ± 0.00	100.00 ± 0.00
F7	100	65.70 ± 15.66	98.20 ± 0.65
F8	97.25	95.98 ± 1.63	97.53 ± 0.05
F9	4.13	1.90 ± 0.78	1.98 ± 0.59
F10	48	80.00 ± 4.29	88.11 ± 1.16
F11	51	31.08 ± 9.33	50.11 ± 3.05
F12	98.63	99.55 ± 0.34	99.85 ± 0.05
F13	94.63	94.03 ± 0.52	94.61 ± 0.26
F14	100	99.88 ± 0.05	99.90 ± 0.05
F15	7.38	2.70 ± 1.21	3.63 ± 1.21
F16	28.63	83.74 ± 3.44	90.46 ± 0.80
F17	84.88	87.55 ± 4.35	94.50 ± 0.86
F18	89.75	89.65 ± 0.33	89.76 ± 0.12
F19	1.75	74.22 ± 9.24	86.25 ± 0.68
F20	47	83.76 ± 9.78	90.78 ± 0.37
F21	37.38	36.70 ± 5.80	45.08 ± 1.58
mean	60.65	68.99 ± 3.99	74.97 ± 1.08

**Table 3:** False alarming rates (FARs) (%) of the TE process normal data sets by SVDD, DeSVDD and EDeSVDD.

No.	SVDD	DeSVDD	EDeSVDD
F1	0	0.97 ± 0.80	0.56 ± 0.55
F2	0	0.84 ± 0.84	0.13 ± 0.26
F3	3.75	2.38 ± 1.77	1.88 ± 1.61
F4	0.63	0.91 ± 0.72	0.44 ± 0.42
F5	0.63	0.91 ± 0.72	0.44 ± 0.42
F6	0	0.41 ± 0.47	0.19 ± 0.30
F7	0	0.63 ± 0.70	0.06 ± 0.20
F8	0	0.81 ± 0.68	0.06 ± 0.20
F9	9.38	2.56 ± 1.62	4.19 ± 1.62
F10	0	0.69 ± 0.64	1.00 ± 0.44
F11	0	0.84 ± 1.04	0.44 ± 0.30
F12	7.5	0.88 ± 1.17	0.94 ± 0.68
F13	1.25	0.47 ± 0.67	0.19 ± 0.42
F14	0	0.94 ± 1.43	0.69 ± 0.46
F15	0	1.56 ± 1.19	0.81 ± 1.02
F16	13.75	2.13 ± 1.02	4.13 ± 1.72
F17	0	1.00 ± 0.71	0.44 ± 0.51
F18	0	0.97 ± 0.89	0.63 ± 0.83
F19	0	0.66 ± 0.74	0.06 ± 0.20
F20	0	0.59 ± 0.92	0.19 ± 0.42
F21	2.5	1.47 ± 1.08	0.88 ± 0.94
mean	1.88	1.08 ± 0.94	0.87 ± 0.64
